# Use of computerized dynamic posturography to assess balance in older adults after nighttime awakenings using zolpidem as a reference

**DOI:** 10.1186/1471-2318-8-15

**Published:** 2008-07-15

**Authors:** Gary Zammit, Sherry Wang-Weigand, Xuejun Peng

**Affiliations:** 1 Clinilabs and the Columbia University College of Physicians and Surgeons, New York, NY, USA; 2 Takeda Global Research & Development Center, Deerfield, IL, USA

## Abstract

**Background:**

Computerized dynamic posturography (CDP) has been used to detect balance and stability impairments in adults of all ages. The goal of the current pilot study was to evaluate balance in healthy older adults after a middle-of-the-night awakening and to assess the ability of CDP to measure effects of bedtime zolpidem administration.

**Methods:**

Two studies used CDP to evaluate balance in healthy older adults (≥ 65 years) during middle-of-the-night awakenings. The first study used a drug-free, single-period, within-subject, repeated measures study design. Subjects were evaluated during the day, pre-sleep, and 2 hours after bedtime for dynamic standing balance using the NeuroCom EquiTest Sensory Organization Test (SOT). Pairwise comparisons were made using one-way ANOVA. The second study was a single-blind, randomized, placebo-controlled, crossover study evaluating the ability of the SOT to measure medication-induced dynamic standing balance impairments using the commonly prescribed sleep medication, zolpidem 10 mg, as a test medication. Assessments were performed at night before zolpidem administration and then again 2 hours after bedtime. Comparisons were made between the 2 groups using an ANCOVA model.

**Results:**

Twelve older adults (mean age 68.4 years) were evaluated in the first study. There was no significant difference between pre-sleep and middle-of-the-night assessments for the SOT composite score (*P *= 0.439). Eleven older adults (mean age 68.9 years) were evaluated in the second study. Zolpidem administration significantly decreased the SOT composite score after a middle-of-the-night awakening compared with placebo (*P *< 0.001).

**Conclusion:**

In healthy older adults, getting up in the middle of the night did not have a significant effect on dynamic standing balance; however, bedtime administration of zolpidem 10 mg did lead to significant impairments. Thus, the SOT was able to measure medication-induced dynamic standing balance impairments and may be useful for future studies comparing balance effects of medications.

## Background

Older adults often take medications to treat a variety of conditions, many of which can profoundly affect balance and stability.[1] Impaired balance and mobility in older adults is particularly concerning since potential consequences include an increased risk of falls and injuries.[2] Many falls in older adults can be attributed to medication-induced balance impairments. [3-5] Additionally, medications with the potential for balance impairments taken at night may pose a significant risk for older adults since approximately 20% of all falls are reported to occur at night.[3]

Several classes of medication commonly prescribed to older adults have demonstrated potential effects on balance, including the sedative-hypnotics (for insomnia), antidepressants (for depression and anxiety), and anticonvulsants (for seizure disorders). In older adults, several studies have suggested that sedative-hypnotics may be associated with impaired balance and an increased risk of falls and hip fractures. [5-9] A retrospective case-control study found that hip fractures occurred twice as often in adults over the age of 65 taking zolpidem compared with those taking no insomnia medication.[8] However, other studies have found no increased risk for falls or hip fractures in older adults taking sedative-hypnotics and attribute balance impairments to insomnia itself.[10] Antidepressants also have been associated with an increased risk of falls in older adults with no significant differences detected between the older tricyclic antidepressants and the newer selective serotonin reuptake inhibitors.[11] Common side effects of anticonvulsants include dizziness and ataxia, which may lead to impaired balance in older adults. In a study comparing the effects of lamotrigine, carbamazepine, and gabapentin on balance, older adults scored below normal on multiple balance assessments.[12]

Most medications are not routinely assessed for the possibility of affecting balance and stability; however, as stated previously, the consequences of impaired balance can be severe in older adults. A test is needed that can detect a medication's relative risk of balance impairment in older adults. This would help to better identify appropriate medications for those patients at particular risk for complications due to potential mobility issues.

Computerized dynamic posturography (CDP) has been used previously to assess balance in a variety of conditions. [12-14] One specific CDP test is the NeuroCom EquiTest Sensory Organization Test (SOT), which provides an extremely sensitive objective assessment of the main sensory systems involved in balance and stability. The SOT was chosen for these studies because it is a well-recognized assessment tool that has been used extensively to evaluate dynamic standing balance both in research and clinical practice. [13-16]

In the current analysis, 2 separate pilot studies used the SOT assessment to evaluate dynamic standing balance in older adults during middle-of-the-night awakenings. In the first study, normal healthy older adults were evaluated after a middle-of-the-night awakening. The goal was to establish a comparative normal balance baseline for further studies in older adults using the SOT assessment and to determine if getting out of bed in the middle of the night was itself a risk factor for balance impairment. The second study was placebo-controlled and evaluated the ability of the SOT to determine medication-induced dynamic standing balance impairments. Zolpidem is a commonly prescribed medication that has been previously determined to alter balance up to 5 hours after administration,[9] and was therefore chosen as the test medication.

## Methods

All studies were conducted in compliance with Good Clinical Practices and applicable Federal and local laws with Institutional Review Board approval. Informed consent was obtained from all subjects prior to enrollment in the study.

### Balance assessment test (EquiTest computerized dynamic posturography)

**Figure 1:**
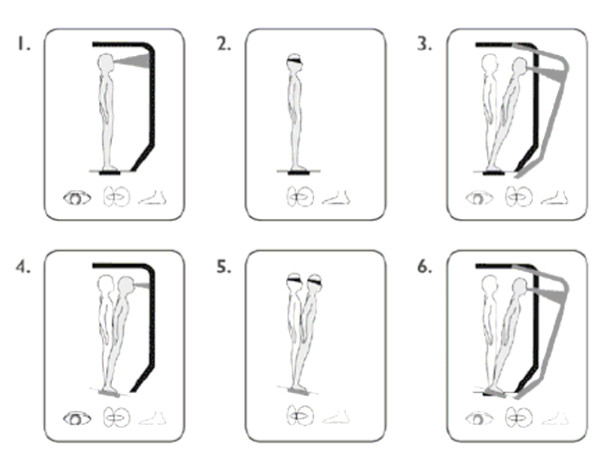
**Six Conditions of the EquiTest Sensory Organization Test Assess Postural Stability and Potential for Falls**. Figure Used Courtesy of NeuroCom^® ^International, Inc.

The NeuroCom EquiTest SOT (NeuroCom International, Inc., Clackamas, OR) assesses postural stability and the potential for falls by identifying abnormalities in the subject's use of the somatosensory, visual, and vestibular sensory systems that contribute to balance. The SOT procedure requires subjects to stand on a pressure-sensitive, dynamic tilted force plate facing a sway-referenced visual surround while strapped into a safety harness to prevent injury in the event of a loss of balance. Each test comprises 3 trials for each of 6 conditions representing different aspects of balance (Figure 1). For condition 1, the subject's eyes are open, and the force plate remains in a fixed position. This condition assesses baseline postural stability under normal circumstances. For condition 2, the subject's eyes are closed, and the force plate remains in a fixed position. For condition 3, the subject's eyes are open, the force plate remains in a fixed position, and the visual surround is tilted. For condition 4, the subject's eyes are open, the force plate tilts, and the visual surround remains upright. For condition 5, the subject's eyes are closed, and the force plate tilts. For condition 6, the subject's eyes are open, the force plate tilts, and the visual surround tilts. For each condition an equilibrium score (ES1-6) is calculated that quantifies the center of gravity sway or postural stability under each of the 3 trials of the 6 sensory conditions. The scores are based on the amount of anterior-posterior sway compared to the maximal theoretical sway limits of stability (8.5° anterior and 4° posterior). The score is calculated by the following formula: ES = {12.5° - (θ_max _- θ_min_)}/12.5° × 100%. In this formula, θ_max _indicates the greatest anterior-posterior sway displayed by the subject and θ_min _indicates the least anterior-posterior sway. A score of 100 represents perfect balance (no sway), and a score of 0 represents a potential fall (sway exceeds limits of stability). If at any time during the test, the subject takes a step or requires the assistance of the safety harness, the subject scores a 0 for that test. An average score is calculated for each of the 6 conditions and a composite equilibrium score (CES) is calculated as a weighted average of all 6 individual scores with each of the first 2 conditions carrying a weight of 1/14 and each of the other 4 conditions carrying a weight of 3/14. These weights were specified by the manufacture to reflect the difficulty levels of the 6 tasks. Sensory analysis ratios also are used to identify possible impairments of individual sensory systems. The somatosensory ratio (condition 2/condition 1), visual ratio (condition 4/condition 1), and vestibular ratio (condition 5/condition 1) assess the ability to use input from each sensory system to control balance. The preference ratio (condition 3 + 6/condition 2 + 5) assesses the extent upon which a subject relies on visual input to control balance, even when the visual information is incorrect. The SOT has been used in many previous studies and has been shown to be a valid assessment of balance impairment in older adults and accurately predicts fall risk.[13,14,17]

### Balance assessment of healthy older adults after a middle-of-the-night awakening

This study evaluated CDP balance assessments using a drug-free, single-period, within-subject, repeated measures study design. Subjects eligible for the study were healthy adults, aged 65 or older, with no history of insomnia, psychiatric disorder, significant medical condition (unless controlled with protocol-allowed medication), or balance impairment. Subjects were asked about any history of recurrent falls, recent serious head injuries, presence of an inner ear or vestibular system disorder, history of dizziness, or any significant visual acuity or field abnormalities not improved with the use of corrective lenses to detect any possible undiagnosed balance impairments. Subjects were not allowed to participate if they had taken any medications known to affect sleep-wake functions or alter the central nervous system, or if they participated in a clinical trial of an investigational drug within 1 week (or 5 half-lives) of the start of the study. Subjects were screened 4–10 days prior to testing, which included documentation of medical history, sleep history (including habitual bedtime), and a physical examination. Subjects were also exposed to the EquiTest CDP tests to acclimate them to the testing procedures and assess their daytime SOT scores. On the day of testing, subjects reported to the clinic 2 hours prior to habitual bedtime and were allowed to relax in a private room. After 30 minutes, a pre-sleep assessment of the SOT was performed as described above. Subjects went to sleep at their habitual bedtime. After 2 hours, subjects were awakened and completed the SOT procedure again. Each SOT assessment consisted of 3 trials for each condition. Following middle-of-the-night testing, subjects returned to bed and were allowed to sleep until morning (8 hours after bedtime). Subjects left the clinic in the morning after a brief neurological exam.

Descriptive statistics were used to summarize the SOT results at daytime, pre-sleep, and after the middle-of-the-night awakening. Pairwise comparisons were made using one-way ANOVA with the time of day as a class effect. A *P *value less than 0.05 was considered statistically significant.

### Effect of zolpidem on balance assessment of healthy older adults after a middle-of-the-night awakening

This study evaluated the ability of CDP to detect medication-induced balance impairments using a single-blind, randomized, placebo-controlled, crossover study design with zolpidem as the test medication. Healthy older adults (≥ 65 years of age) with no history of sleep or balance disorders were included in this study. The subjects enrolled in this study were not the same as those enrolled in the first, drug-free study. Subjects were excluded from the study if they had a history of a psychiatric disorder or significant medical condition (unless controlled with protocol-allowed medication), had taken any medications known to affect sleep-wake functions or alter the central nervous system, or if they participated in a clinical trial of an investigational drug within 1 week (or 5 half-lives) of the start of the study. Subjects with a history of recurrent falls, recent serious head injuries, presence of an inner ear or vestibular system disorder, history of dizziness, or any significant visual acuity or field abnormalities not improved with the use of corrective lenses were excluded from the study. At initial screening (between Days -7 and -1), medical histories were obtained, and subjects underwent a physical exam, 12-lead electrocardiogram, and CDP assessments. On the first crossover treatment day (Day 1), subjects arrived at the clinic 2 hours before habitual bedtime, and a pre-sleep assessment for the SOT was obtained. Immediately prior to bedtime, subjects were randomly assigned to receive either zolpidem 10 mg or placebo. Subjects were awakened after 2 hours for middle-of-the-night SOT balance assessment. After balance testing, subjects were allowed to go back to sleep until morning (8 hours after bedtime). Subjects were required to remain at the clinic until noon to assess potential residual sedation. On the second crossover treatment day (between Days 4 and 7), subjects followed the same procedures and were assigned to receive the opposite treatment as their first visit (zolpidem 10 mg or placebo). Adverse events (AEs) were monitored throughout the study.

Descriptive statistics were used to summarize the SOT results for the placebo and zolpidem 10 mg groups. Comparisons were made between the 2 groups using an ANCOVA with sequence, period, and treatment group as class effects and the pre-treatment baseline measurement as a continuous covariate. A *P *value less than 0.05 was considered statistically significant.

## Results

### Balance assessment of healthy older adults after a middle-of-the-night awakening

A total of 12 older adults (1 man, 11 women, age range 65 – 80 years, average age 68.4 years) completed the study. Results for the SOT assessment are shown in Table 1. There was no significant difference between the daytime or pre-sleep assessments and the middle-of-the-night assessment for the composite equilibrium score or any of the individual equilibrium scores for the SOT. There were also no detected impairments in any of the ratios (somatosensory, visual, vestibular, or preference) designed to evaluate sensory system function.

**Table 1:** SOT Assessments in Healthy Older Adults during the Day, Pre-Sleep, and after a Middle-of-the-Night Awakening

**SOT Assessment**	Daytime* (mean ± SE)	Pre-sleep* (mean ± SE)	Middle-of-the-Night* (mean ± SE)	**Daytime vs Middle-of-the-Night *P *value**	**Pre-sleep vs Middle-of-the-Night *P *value**
ES1 (%)	92.84 ± 0.72	94.06 ± 0.86	93.44 ± 0.86	0.594	0.617
ES2 (%)	92.27 ± 0.59	92.33 ± 0.70	92.61 ± 0.70	0.714	0.780
ES3 (%)	89.73 ± 1.29	93.22 ± 1.54	92.00 ± 1.54	0.265	0.578
ES4 (%)	76.00 ± 2.36	83.08 ± 2.81	79.39 ± 2.81	0.362	0.358
ES5 (%)	59.00 ± 3.04	65.83 ± 3.62	66.58 ± 3.62	0.117	0.884
ES6 (%)	52.31 ± 3.22	65.50 ± 3.83	60.36 ± 3.83	0.116	0.349
CES(%)	72.53 ± 1.52	79.25 ± 1.81	77.25 ± 1.81	0.053	0.439
Somatosensory Ratio (%)	99.47 ± 0.54	98.25 ± 0.64	99.25 ± 0.64	0.794	0.279
Visual Ratio (%)	81.94 ± 2.50	88.58 ± 2.98	85.17 ± 2.98	0.412	0.422
Vestibular Ratio (%)	63.71 ± 3.25	70.17 ± 3.87	71.25 ± 3.87	0.144	0.844
Preference Ratio (%)	94.41 ± 2.33	100.67 ± 2.77	95.92 ± 2.77	0.680	0.233

*Scores are within normal ranges for adults ≥ 65 years old.[13,14,20]

ES = equilibrium scores

CES = composite equilibrium score

### Effect of zolpidem on balance assessment of healthy older adults after a middle-of-the-night awakening

A total of 11 older adults (3 men, 8 women, age range 65 – 81 years, average age 68.9 years) were enrolled in the crossover study, with 1 subject discontinuing after the first treatment period (only completed zolpidem 10 mg treatment). Pre-sleep baseline assessments (before medication administration) showed no significant differences between the placebo and zolpidem groups on any of the SOT equilibrium scores or SOT sensory ratios (*P *> 0.05 for both) and were within normal limits for older adults. The differences between the placebo and zolpidem middle-of-the-night SOT assessment is shown in Table 2. Bedtime zolpidem administration resulted in a significant decrease in the composite equilibrium score after a middle-of-the-night awakening compared with placebo (*P *< 0.001). The vestibular ratio was also significantly decreased after zolpidem treatment compared with placebo (*P *< 0.002).

**Table 2:** SOT Assessments Comparing Zolpidem 10 mg and Placebo after a Middle-of-the-Night Awakening

**SOT Assessment**	**Difference Between Zolpidem 10 mg and Placebo (LS mean ± SE)**	***P *value**
ES1 (%)	-6.67 ± 2.02	0.013
ES2 (%)	-7.41 ± 1.90	0.006
ES3 (%)	-6.35 ± 2.78	0.056
ES4 (%)	-13.14 ± 3.76	0.010
ES5 (%)	-27.69 ± 5.38	0.001
ES6 (%)	-17.21 ± 3.61	0.002
CES (%)	-15.83 ± 2.88	<0.001
Somatosensory Ratio (%)	-1.74 ± 1.60	0.315
Visual Ratio (%)	-8.76 ± 4.00	0.065
Vestibular Ratio (%)	-27.43 ± 5.91	0.002
Preference Ratio (%)	12.82 ± 5.90	0.066

ES = equilibrium scores

CES = composite equilibrium score

Out of 11 subjects, 1 AE occurred during the placebo phase (headache) and 2 AEs occurred during the zolpidem phase (upper respiratory infection and headache). The 2 occurrences of headache were deemed possibly related to study drug while the upper respiratory infection was deemed unrelated to study drug. All AEs were considered mild.

## Discussion

The majority of balance studies in older adults have focused on daytime assessments of stability and fall risk.[18,19] The current study evaluated balance and stability during the night in healthy older adults with and without administration of a sedative-hypnotic before bed.

Using the NeuroCom EquiTest SOT balance assessment, healthy older adults exhibited no significant impairments after a middle-of-the-night awakening compared with pre-sleep measurements. This suggests that getting out of bed in the middle of the night, by itself, does not affect dynamic standing balance in healthy older adults with no history of impaired balance. Any increase in balance problems or falls during the night in older adults might therefore indicate an underlying medical disorder or a symptom of nighttime medication effects on balance rather than a symptom of aging. However, the small sample size in this study may have prevented the detection of small balance impairments. Also, the SOT is only designed to measure postural control and the sensory systems associated with standing balance. It is possible that other aspects of balance may be affected after a middle-of-the-night awakening. The daytime assessments were also obtained during the screening period and may be lower due to unfamiliarity with the SOT procedures. Further assessments comparing daytime and nighttime balance may be needed to fully understand the time-of-day effects on balance in older adults.

In contrast to the first pilot study, dynamic standing balance was significantly impaired during the middle of the night after a bedtime dose of zolpidem 10 mg in the second study. This suggests that the SOT has the ability to measure medication-induced dynamic standing balance impairments, since previous studies using other measures of balance have shown significant balance impairments and increased fall risk after zolpidem administration.[8,9,20] Even with the small number of subjects in the current study, significant middle-of-the-night balance impairments were detected after zolpidem administration, suggesting that zolpidem would be a good positive control for future treatment comparison balance studies using the SOT.

Balance was evaluated in the current study using the NeuroCom EquiTest SOT assessment. Several studies have demonstrated the association between impaired SOT equilibrium scores and the incidence of falls in adults of all ages.[13,14,21,22] In healthy adults (mean age 45), the incidence of multiple falls within a 3-year period was significantly associated with lower SOT equilibrium scores.[13] Similarly, in older adults, SOT composite scores were significantly lower in subjects with a history of falls compared to non-fallers.[21] The combined evidence from these and other studies suggests that balance assessment using the EquiTest SOT is a strong predictor of fall risk.

## Conclusion

In healthy older adults, getting up in the middle of the night did not have any significant effect on dynamic standing balance; however, bedtime administration of zolpidem 10 mg did lead to significant deficits. The SOT assessment successfully detected dynamic standing balance impairments due to zolpidem and may be useful for future studies comparing balance effects of medications. However, additional measures may be useful in assessing other aspects of balance to gain a fuller understanding of the effects of medications on balance.

## Competing interests

This study was supported by the Takeda Pharmaceutical Company, Ltd., Osaka, Japan. SWW and XP are employees of the Takeda Global Research and Development Center. GZ has received grants/research support or worked as a consultant for Ancile Pharmaceuticals, Arena, Aventis, Biovail, Boehringer-Ingelheim, Cephalon Inc., Elan, Eli Lilly, Epix, Evotec, Forest, Glaxo Smith Kline, Jazz, King Pharmaceuticals, Ligand, H. Lundbeck A/S, McNeil, Merck and Co., National Institutes of Health (NIH), Neurim, Neurocrine Biosciences, Neurogen, Organon, Orphan Medical, Pfizer, Renovis, Respironics, Sanofi-Aventis, Sanofi-Synthelabo, Schering-Plough, Select Comfort, Sepracor, Shire, Somaxon, Somnus, Takeda Pharmaceuticals North America, Transcept, UCB Pharma, Predix, Vanda, Vela, and Wyeth-Ayerst Research. GZ is also a shareholder of Clinilabs, Inc.

## Authors' contributions

SW–W and XP are employees of Takeda Global Research and Development Center and participated in the study design, data analysis, and interpretation of results. GZ was involved in the study design, data analysis, and interpretation of results. GZ, SW–W, and XP all participated fully in the writing and review of the manuscript.

## Pre-publication history

The pre-publication history for this paper can be accessed here:



## References

[B1] Leipzig RM, Cumming RG, Tinetti ME (1999). Drugs and falls in older people: a systematic review and meta-analysis: I. Psychotropic drugs. J Am Geriatr Soc.

[B2] Agostini JV, Han L, Tinetti ME (2004). The relationship between number of medications and weight loss or impaired balance in older adults. J Am Geriatr Soc.

[B3] Masud T, Morris RO (2001). Epidemiology of falls. Age Ageing.

[B4] Agostini JV, Tinetti ME (2002). Drugs and falls: rethinking the approach to medication risk in older adults. J Am Geriatr Soc.

[B5] Allain H, Bentue-Ferrer D, Polard E, Akwa Y, Patat A (2005). Postural instability and consequent falls and hip fractures associated with use of hypnotics in the elderly: a comparative review. Drugs Aging.

[B6] Wagner AK, Zhang F, Soumerai SB, Walker AM, Gurwitz JH, Glynn RJ (2004). Benzodiazepine use and hip fractures in the elderly: who is at greatest risk?. Arch Intern Med.

[B7] Nurmi-Luthje I, Kaukonen JP, Luthje P, Naboulsi H, Tanninen S, Kataja M (2006). Use of benzodiazepines and benzodiazepine-related drugs among 223 patients with an acute hip fracture in Finland: Comparison of benzodiazepine findings in medical records and laboratory assays. Drugs Aging.

[B8] Wang PS, Bohn RL, Glynn RJ, Mogun H, Avorn J (2001). Zolpidem use and hip fractures in older people. J Am Geriatr Soc.

[B9] Allain H, Bentue-Ferrer D, Tarral A, Gandon JM (2003). Effects on postural oscillation and memory functions of a single dose of zolpidem 5 mg, zopiclone 3.75 mg and lormetazepam 1 mg in elderly healthy subjects. A randomized, cross-over, double-blind study versus placebo. Eur J Clin Pharmacol.

[B10] Avidan AY, Fries B, James M, Szafara K, Wright G, Chervin R (2005). Insomnia and hypnotic use, recorded in the minimum data set, as predictors of falls and hip fractures in Michigan nursing homes. J Am Geriatr Soc.

[B11] Ensrud KE, Blackwell TL, Mangione CM, Bowman PJ, Whooley MA, Bauer DC (2002). Central nervous system-active medications and risk for falls in older women. J Am Geriatr Soc.

[B12] Fife TD, Blum D, Fisher RS (2006). Measuring the effects of antiepileptic medications on balance in older people. Epilepsy Research.

[B13] Vouriot A, Gauchard GC, Chau N, Benamghar L, Lepori ML, Mur JM (2004). Sensorial organisation favouring higher visual contribution is a risk factor of falls in an occupational setting. Neurosci Res.

[B14] Whitney SL, Marchetti GF, Schade AI (2006). The relationship between falls history and computerized dynamic posturography in persons with balance and vestibular disorders. Arch Phys Med Rehabil.

[B15] Guskiewicz KM, Riemann BL, Perrin DH, Nashner LM (1997). Alternative approaches to the assessment of mild head injury in athletes. Med Sci Sports Exerc.

[B16] Ray C, Horvat M, Croce R, Christopher MR, Wolf S (2007). The impact of vision loss on postural stability and balance strategies in individuals with profound vision loss. Gait & Posture.

[B17] Cohen H, Heaton L, Congdon S, Jenkins H (1996). Changes in sensory organization test scores with age. Age Ageing.

[B18] Woolley SM, Czaja SJ, Drury CG (1997). An assessment of falls in elderly men and women. J Gerontol A Biol Sci Med Sci.

[B19] Camicioli R, Panzer VP, Kaye J (1997). Balance in the healthy elderly: posturography and clinical assessment. Arch Neurol.

[B20] Evans SM, Funderburk FR, Griffiths RR (1990). Zolpidem and triazolam in humans: behavioral and subjective effects and abuse liability. J Pharmacol Exp Ther.

[B21] Wallmann HW (2001). Comparison of elderly nonfallers and fallers on performance measures of functional reach, sensory organization, and limits of stability. J Gerontol A Biol Sci Med Sci.

[B22] Forizetti P, Panzer V, Reding M (2000). Use of computerized dynamic posturography in the assessment of elderly fallers. Neurorehabilitation and Neural Repair.

